# Ectopic parathyroid carcinoma coexisting with Graves’ disease: A case report and literature review

**DOI:** 10.1097/MD.0000000000048800

**Published:** 2026-05-15

**Authors:** Gui-Zhi Li, Na Li, Ze-Ping Wang, Wen-Yu Tang, Zhan-Sheng Zhao

**Affiliations:** a Department of Endocrinology, The Second Hospital of Hebei Medical University, Shijiazhuang, Hebei, People’s Republic of China.

**Keywords:** ectopic parathyroid, Graves’ disease, hypercalcemia, hyperthyroidism, parathyroid carcinoma, parathyroid hormone

## Abstract

**Rationale:**

Parathyroid carcinoma (PC) is a rare malignancy typically characterized by severe hypercalcemia and markedly elevated parathyroid hormone (PTH). The coexistence of PC with Graves’ disease (GD) is exceptionally rare and presents a critical diagnostic challenge. The symptoms of hyperthyroidism can mask the clinical features of parathyroid disease, and when PC presents with only mild biochemical abnormalities – as in this case – the risk of misdiagnosis or delayed treatment is significantly increased. This case is reported to highlight the deceptive nature of this combination and the necessity for vigilance even when laboratory values are not drastically elevated.

**Patient concerns:**

A 56-year-old patient presented with clinical manifestations of hyperthyroidism and a palpable left-sided neck mass. Crucially, the patient lacked the classic red flags of PC, such as severe bone pain, renal colic, or hypercalcemic crisis, which typically prompt immediate investigation for malignancy.

**Diagnoses:**

Initial laboratory evaluations revealed hyperthyroidism consistent with Graves’ disease. However, calcium and parathyroid hormone (PTH) levels were only mildly elevated, contrasting with the markedly high levels typically seen in carcinoma. A computed tomography scan identified a substantial mass located between the left carotid sheath and the thyroid gland, suggesting an ectopic lesion. Post-surgical pathological examination definitively confirmed the diagnosis of PC.

**Interventions:**

Given the presence of the neck mass and biochemical abnormalities, the patient underwent surgical resection of the tumor.

**Outcomes:**

The surgery was successful with no immediate complications. Postoperatively, the serum calcium and PTH levels returned to normal ranges rapidly. The patient recovered without complications, and follow-up has shown no signs of recurrence.

**Lessons:**

This case highlights the diagnostic challenge of PC when masked by Graves’ disease and presenting with atypical, mild biochemical elevations. Clinicians should maintain a high index of suspicion for parathyroid malignancy in patients with Graves’ disease who present with a neck mass, even if calcium and PTH levels do not reach the extreme thresholds typically associated with carcinoma. Early identification and radical resection are crucial for favorable prognosis.

## 1. Introduction

Parathyroid carcinoma is one of the rarest cancers, accounting for 0.005% of all cancers and <1% of cases of primary hyperparathyroidism.^[1,2]^ PC is typically sporadic and occurs less frequently in cases of genetic syndromes, such as hyperparathyroidism-jaw tumor syndrome and multiple endocrine tumors (MENs).^[3]^ Clinically, PC is typically distinguished from benign adenomas by a classic presentation of severe hypercalcemia, markedly elevated parathyroid hormone (PTH) levels (often >3–10 times the upper limit of normal), and palpable neck masses.^[4]^ However, the diagnostic landscape becomes profoundly complex when PC coexists with Graves’ disease, a dual pathology that presents a critical diagnostic challenge.

A search of PubMed from 1960 to the present day using the terms “parathyroid carcinoma” and “thyrotoxicosis, Graves’ disease” revealed only 4 cases.^[5–8]^ Beyond its rarity, this combination is clinically deceptive because the symptoms of hyperthyroidism – such as weight loss, palpitations, and tremors – can effectively mask the clinical features of parathyroid disease. Furthermore, because Graves’ disease itself can induce mild hypercalcemia through increased bone resorption, clinicians may attribute elevated calcium levels solely to thyroid toxicity, potentially delaying the investigation of a concomitant parathyroid malignancy.

In this report, we present a case that challenges even these complex diagnostic paradigms. unlike the few previously reported cases which exhibited classic severe biochemical derangements, our patient presented with a massive ectopic parathyroid carcinoma but only mild biochemical abnormalities. This functional silence of a large, aggressive tumor, combined with the masking effect of Graves’ disease, highlights a significant diagnostic pitfall. Therefore, this study aims to elucidate the “masking effect” of Graves’ disease on parathyroid carcinoma and underscores the necessity for a high index of suspicion for malignancy in patients presenting with neck masses, even when calcium and PTH levels do not reach the extreme thresholds typically associated with carcinoma.

## 2. Statistical analysis

Descriptive statistics were employed to summarize the clinical and biochemical characteristics of the identified cases from the literature review. Continuous variables, including age, serum calcium levels, intact parathyroid hormone (PTH) levels, and tumor maximum diameter, were expressed as mean ± standard deviation and range (minimum–maximum). Categorical variables, such as sex and anatomical location, were presented as frequencies and percentages. All data processing and descriptive analyses were performed using Microsoft Excel 2021.

## 3. Case presentation

### 3.1. Diagnostic assessment

A 56-year-old woman presented to the Department of Endocrinology of the Second Hospital of Hebei Medical University on April 11, 2024, complaining of palpitations, hand tremors, and weight loss for 2 months. During the past 2 months, she had lost approximately 5 kg in weight but did not seek treatment. Two days prior, she was diagnosed with hyperthyroidism at a local hospital and started taking 12.5 mg of oral metoprolol tartrate twice daily with no symptom relief. One day prior, she presented at our clinic and was diagnosed with Graves’ disease (GD) due to thyroid function. Thyroid function testing revealed the following: free triiodothyronine (FT3) >30.8 pmol/L (normal range, 2.3–6.8 pmol/L), serum free thyroid hormone (FT4) >154 pmol/L (normal range, 10–23.5 pmol/L), thyroid stimulating hormone <0.008 mU/L (normal range, 0.34–4.0 mU/L), TPOAb >1300 IU/L, and TRAb >40 IU/L. The biochemical test results revealed an ALP concentration of 206.1 U/L (reference range 50–135 U/L), an ALT concentration of 43.8 U/L (reference range 7–40 U/L), an AST concentration of 38.7 U/L (reference range 13–35 U/L), and a 25-OH-D concentration of 25.5 nmol/L (reference range ≥50 nmol/L). A thyroid ultrasound revealed a diffuse thyroid change with abundant blood flow and a solid hypoechoic nodule on the left lateral neck measuring 5.93 × 2.51 × 2.47 cm. This mass exhibited distinct boundaries and contained small echogenic foci internally. The possibility of an ectopic thyroid was taken into consideration. The patient provided a negative response on the presence of symptoms, such as hoarseness, breathing trouble, dizziness, or loss of strength. She also denied a history of kidney disease and other similar diseases. The patient’s family requested surgical treatment for the neck mass. For further evaluation and treatment, the patient was transferred to the general surgery department for surgical intervention.

**Figure 1.**
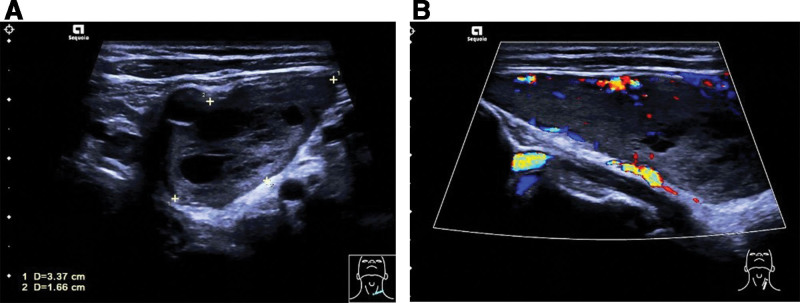
Ultrasound of the neck showing a huge mixed cystic and solid mass (A) in the left supraclavicular region (partially behind the common carotid artery) and vascularity (B).

**Figure 2.**
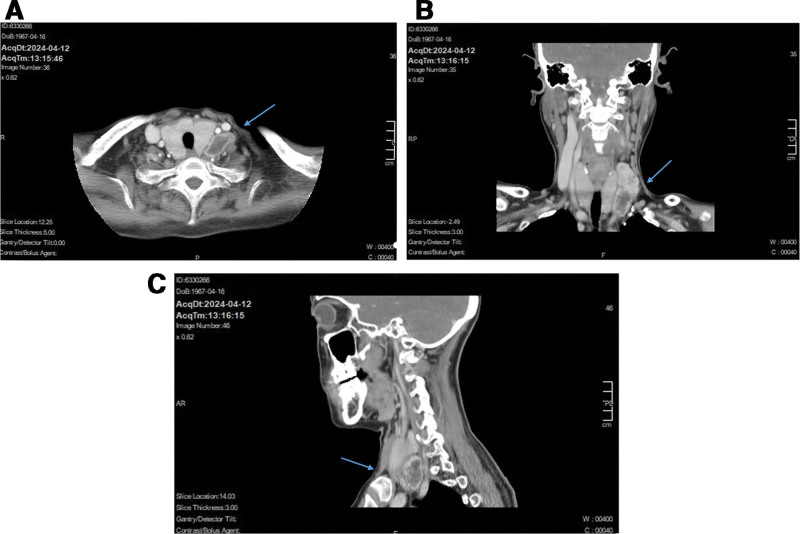
CT examinations. (A) Enhanced CT scan of the neck in horizontal position. (B) Enhanced CT scan of the neck in coronary position. (C) Enhanced CT scan of the neck in sagittal position. The arrows indicate the large cystic-solid mass (parathyroid carcinoma) located between the left carotid sheath and the thyroid gland.

Physical examination revealed a height of 155 cm, weight of 58.5 kg, and body mass index of 24.3 kg/m^2^. The patient looked well and was in no pain, distress, or discomfort. There was no tremor or hoarseness. The patient’s pulse rate was 102 beats per minute, with a blood pressure of 123/70 mm Hg. A diffuse goiter was observed, and a vascular murmur could be heard. The laboratory findings on admission revealed normal renal function, a mild degree of hypercalcemia (Ca) (2.71 mmol/L vs normal range: 2.11–2.52 mmol/L), normal phosphate, and increased levels of intact parathyroid hormone (PTH) (14.7 pmol/L vs normal range: 1.59–6.86 pmol/L). A repeat neck ultrasonography examination revealed the presence of a large mixed cystic and solid mass measuring 5.97 × 3.37 × 1.66 cm in the left supraclavicular area (partially behind the common carotid artery). Blood flow was visible around the mass (Fig. 1). Although these USG findings initially gave the impression of a complex thyroid mass due to the background of diffuse goiter, the distinct boundaries observed warranted further investigation. A computed tomography (CT) scan was subsequently performed to define the anatomical relationships. A CT scan of the neck revealed the presence of a substantial mass located on the left side. The tumor was located between the left carotid sheath and the left lobe of the thyroid, with no apparent connection to the thyroid gland. It caused compression displacement of the thyroid toward the right (Fig. 2A–C).

### 3.2. Treatment

**Figure 3.**
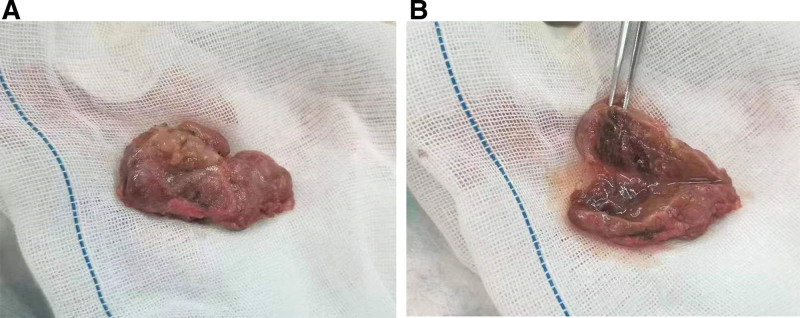
Surgery images. (A) Appearance of the tumor after resection. (B) Image of the surgical cavity after tumor resection.

**Figure 4.**
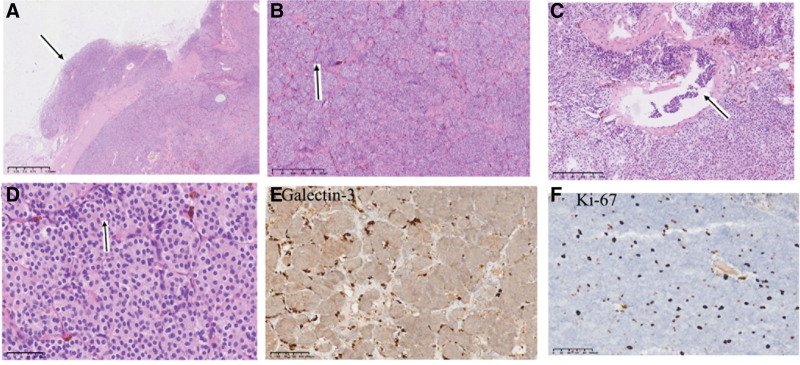
histopathological and immunohistochemical examinations. (A) The tumor has invaded outside the capsule. (H&E X 400). (B) Photomicrograph featuring tumor nests within fibrotic stroma surrounded by clusters of tumor cells (H&E X 400). (C) vascular invasion in the soft tissue surrounding the parathyroid (H&E X 200). (D) Pathological mitotic figures can be seen within the tumor cells (H&E X 50).(E) Gal × 200. (F) Ki-67 × 100.

Definitive Diagnosis According to WHO Criteria, The diagnosis of parathyroid carcinoma was definitively established only upon postoperative histopathological examination. Given the large neck mass and confirming thyroid dysfunction, the patient was transferred to the Department of General Surgery. During the operation, a large left tumor was found, intimately involving the left carotid sheath. Pathology of the frozen section of this material revealed a follicular tumor of the thyroid. A surgical procedure, which included removal of the left thyroid lobe and isthmus, subtotal right thyroidectomy, and excision of the left neck mass, was performed. The dimensions of the mass were measured to be 6.0 × 3.0 × 2.0 cm (Fig. 3A and B). Postoperative pathology of the mass revealed capsule invasion in some areas, local vascular invasion, fibrous septa dividing the tumor cells into nests of varying sizes, and mitotic figures in the tumor cells (Fig. 4A–D). Immunohistochemical analysis revealed robust Galectin-3 expression and a Ki-67 proliferation index of 7% (Fig. 4E and F). These definitive signs of invasion confirmed the diagnosis of ectopic parathyroid carcinoma, correcting the initial clinical impression of a benign thyroid or parathyroid lesion.

### 3.3. Outcome and follow-up

Postoperatively, the patient’s serum calcium and PTH levels normalized rapidly. The dynamic changes in key biochemical markers before and after surgery are summarized in (Table 1.) On the first day postsurgery, the PTH level decreased precipitously to 1.36 pmol/L, and the calcium level dropped to 1.77 mmol/L. At the 1- and 2-month follow-up visits, both parameters stabilized within the normal reference ranges. Given these findings, further therapy was not pursued.

**Table 1:** Comparison of clinical and biochemical parameters before and after surgical intervention.

Parameters	Preoperative	Postoperative (day 1)	Follow-up (1–2 mo)	Normal range
Biochemical markers				
Serum calcium (mmol/L)	2.71	1.77	2.15	2.11–2.52
Intact PTH (pmol/L)	14.7	1.36	3.62	1.59–6.86
25-hydroxyvitamin D (ng/mL)	12.37	N/R	28.50	30.0–100.0
Free T3 (pmol/L)	>30.8	N/R	4.52	2.3–6.8
Free T4 (pmol/L)	>154.0	N/R	15.6	10.0–23.5
TSH (mU/L)	<0.008	N/R	1.25	0.34–4.0
Clinical status				
Neck mass	Present (5.97 cm)	Removed	No recurrence	Absent
Symptoms	Palpitations, tremors	Relieved	Asymptomatic	Absent

FT3 = free triiodothyronine, FT4 = free thyroxine, N/R = not reported, PTH = parathyroid hormone, TSH = thyroid stimulating hormone.

## 4. Discussion

Parathyroid carcinoma is one of the rarest cancers, accounting for <1% of primary hyperparathyroidism cases and approximately 0.005% of all cancers.^[9]^ The incidence of giant parathyroid carcinoma remains unknown.^[10]^ The coexistence of PC and Graves’ disease is an exceptionally rare clinical entity that poses significant diagnostic challenges.^[8]^ While primary hyperparathyroidism (PHPT) frequently co-occurs with thyroid disorders, the specific combination of PC and Graves’ disease has been reported in only a few cases since 1960.^[11]^ A search of PubMed using the terms “parathyroid carcinoma” and “thyrotoxicosis, Graves’ disease” from 1960 to the present returned only 5 cases (including the present case), with common clinical features including severe hypercalcemia, multiple kidney stones, and osteoporosis^[5–8]^ (Table 2). Based on our pooled analysis, the mean age of these patients was 51.25 ± 10.7 years (range 39–65 years), with a 100% female predominance (in cases where sex was reported). The mean tumor size was 3.16 ± 2.5 cm (range 1.2–6.0 cm). Notably, while most cases exhibited severe hypercalcemia (mean 15.3 ± 5.8 mg/dL), our case presented with a relatively mild calcium level (10.84 mg/dL) despite a significantly larger tumor (6.0 cm). PC represents an exceedingly rare malignancy characterized by profound diagnostic challenges and a dearth of systemic therapies, necessitating a multi-modal diagnostic framework that integrates clinical presentation, intraoperative frozen section analysis, and comprehensive histopathological and immunohistochemical evaluation.^[12]^ The present case is distinguished not only by this rare dual pathology but also by its significant departure from the classical clinical hallmarks of PC. In contrast to the typical presentation of severe hypercalcemia and profound PTH elevation, our patient exhibited a biochemical paradox, wherein a massive tumor burden was associated with only disproportionately mild biochemical derangements.

**Table 2:** Clinical and biochemical characteristics of patients with parathyroid carcinoma and concomitant hyperthyroidism reported since 1960.

First author [ref] and year	Age/sex	Thyroid disease	Serum calcium (mg/dL)	PTH (pmol/L)	Tumor size (cm)	Location of PC	Treatment
Current case (2024)	56/F	Graves’ disease	10.84	14.7	6.0	Left carotid sheath (Ectopic)	En-bloc resection
Campennì^[8]^ 2017	65/F	Toxic nodule (AFTN)	10.8	17.0	1.2	Right paratracheal	En-bloc resection
Walton^[7]^ 2015	39/F	Subclinical hyperthyroidism	21.84	70.6	4.3	Right paratracheal	En-bloc resection
Chen^[5]^ 1993	45/F	Thyrotoxicosis	High	High	N/R	N/R	Surgery
Vialettes^[6]^ 1976	N/R	Toxic thyroid adenoma	High	High	N/R	N/R	Surgery

AFTN = autonomously functioning thyroid nodules, F = female, N/R = not reported, PC = parathyroid carcinoma, PTH = parathyroid hormone.

The Deceptive Interaction Between Graves’ Disease and Hyperparathyroidism, the most critical diagnostic pitfall in this case was the confounding physiology of Graves’ disease. Hypercalcemia caused by thyroid toxicity accounts for approximately 47.1%, whereas hypercalcemia caused by Graves’ disease accounts for as many as 73%.^[13]^ To understand why our patient’s presentation was atypical, it is crucial to outline the normal mechanistic framework of the thyrotoxicosis-hypercalcemia-PTH axis. Excess thyroid hormones directly stimulate osteoclastic activity, accelerating bone resorption and releasing calcium into the systemic circulation.^[14,15]^ This resultant hypercalcemia subsequently triggers a negative feedback loop via calcium-sensing receptors on the parathyroid glands, leading to the physiologic suppression of PTH secretion.^[16]^ Consequently, clinicians often attribute mild hypercalcemia in thyrotoxic patients solely to this mechanism, expecting low PTH levels.

However, this case highlights a crucial clinical insight: in the presence of hypercalcemia, a non-suppressed or mildly elevated PTH (14.7 pmol/L in this case) is pathological. While this level might be considered modest for PC, it is disproportionately high for a patient with concomitant thyrotoxicosis, where PTH should theoretically be low. This subtle relative elevation rather than an absolute skyrocketing of PTH is a key indicator that can easily be overlooked.

### 4.1. The biochemical paradox: discordance between tumor size and hormone secretion

A striking feature of this case was the discordance between the massive tumor size (6.0 cm) and the relatively mild biochemical profile. Classically, PC is suspected based on the “3 + 3 rule”: serum calcium > 3 mmol/L (12 mg/dL) and a tumor size > 3 cm. Furthermore, PTH levels in PC are typically 3 to 10 times the upper limit of normal.^[17]^ Studies have indicated that patients with parathyroid carcinoma have higher serum calcium levels than patients with parathyroid adenoma (3.0 mmol/L), with only 10% of parathyroid carcinoma patients having calcium levels <3.0 mmol/L.^[18]^ Our patient, despite having a tumor twice the size of the threshold (6.0 cm), had calcium levels (2.71 mmol/L) well below the typical carcinoma range.

We propose that this paradox is explained by the intrinsic histopathological characteristics of the tumor. One possible explanation for the mild elevation in the patient’s indicators could be intrinsic factors. The tumor tissue of this patient had a large amount of necrosis and cystic changes. Previous studies have indicated that the secretion of PTH by adenomas depends on various factors, not strictly on cell quantity. In very large glandular parathyroid adenoma, the secretion capacity of the cells and the regulatory set point for serum calcium may differ. Additionally, a greater proportion of glandular parathyroid adenomas could have consisted of fibrotic and calcified tissue, with associated hemorrhage and cystic degeneration, leading to a lower rate of PTH secretion per unit of adenoma tissue.^[10]^ The patient’s serum PTH level was 14.7 pmol/L, which may explain the relatively mild elevation in PTH. This functional silence of a large malignancy is deceptive, as it deprives the clinician of the severe biochemical warning signs typically associated with parathyroid cancer.

### 4.2. Ectopic location and the thyroid nodule mimic

The diagnostic complexity was further compounded by the tumor’s ectopic location. Although a preoperative 99mTc-MIBI scan was unfortunately not completed, neck ultrasound, CT, and surgical findings suggested that the parathyroid carcinoma was ectopic in the carotid sheath. Ectopic parathyroid lesions account for 9% to 22% of parathyroid disorders.^[19,20]^ The superior parathyroid glands originate from the dorsal aspect of the fourth pharyngeal pouch and are often found on the posterior surface of the thyroid. Common ectopic locations for superior parathyroid glands include the tracheoesophageal groove, esophageal region, posterior superior mediastinum, paratracheal area, thyroid, and carotid sheath. The inferior parathyroid glands and thymus arise from the third pharyngeal pouch and descend together, with the final location of the inferior parathyroid glands being more variable than that of the superior glands. Common ectopic sites for inferior parathyroid glands include the thymus, thyroid, thyroid ligament, and submandibular area.^[21]^ This case involved an ectopic mass located in the left carotid sheath, likely originating from the superior parathyroid gland. Clinically, a mass in this region, especially in the context of a diffuse goiter and hyperthyroidism, strongly mimics a thyroid nodule or lymphadenopathy rather than a parathyroid lesion. The ultrasound findings of a mixed cystic and solid mass combined with the mild biochemistry could have easily led to a misdiagnosis of a thyroid cyst or adenoma. This underscores the necessity of including parathyroid pathology in the differential diagnosis of lateral neck masses in Graves’ patients, even when the mass appears anatomically distinct from the thyroid bed.

### 4.3. Prognostic significance of immunohistochemical markers

Beyond the structural evidence of invasion, the immunohistochemical profile provides critical insight into the tumor’s biological behavior and prognosis. Galectin-3, which was strongly expressed in our case, is widely recognized as a sensitive marker for distinguishing parathyroid carcinoma from adenoma.^[22]^ While its primary role is diagnostic, its robust expression supports the malignant phenotype of the lesion. More critically, the Ki-67 proliferation index serves as a vital prognostic determinant. According to the consensus statement of the European Society of Endocrine Surgeons, a Ki-67 index >5% is strongly associated with parathyroid malignancy and correlates with a significantly higher risk of local recurrence and distant metastasis.^[12]^ In the present case, the Ki-67 index was 7%, clearly exceeding this high-risk threshold. This finding is pivotal: it suggests that despite the patient’s deceptively mild biochemical profile (the “biochemical paradox”), the tumor possesses a high intrinsic proliferative potential. This elevated Ki-67 index serves as a biological warning, validating the necessity for the radical resection performed and mandating a rigorous, long-term surveillance protocol to monitor for recurrence, even in the absence of severe initial symptoms.

## 5. Conclusion

In summary, this case challenges the prevailing clinical assumption that parathyroid carcinoma must present with severe hypercalcemic crisis. We identify a “triple masking” effect: Graves’ disease symptoms obscured the clinical picture; thyrotoxicosis-associated hypercalcemia provided a false alternative explanation for elevated calcium; and intrinsic tumor necrosis resulted in atypically mild PTH elevation despite the giant tumor size. Furthermore, the elevated Ki-67 index (7%) indicates an aggressive biological potential that belies the mild clinical presentation. This report advocates for a high index of suspicion for parathyroid malignancy in Graves’ disease patients presenting with neck masses and non-suppressed PTH, ensuring that “biochemical silence” does not delay potentially life-saving radical resection.

## Acknowledgments

We are indebted to the cooperation of the patient. We also thank Hao Zeng-fang (Department of Pathology, Second Hospital of Hebei Medical University) for their work in the pathological examination.

## Author contributions

**Conceptualization:** Zhan-Sheng Zhao.

**Data curation:** Ze-Ping Wang, Wen-Yu Tang.

**Investigation:** Ze-Ping Wang, Gui-Zhi Li.

**Software:** Wen-Yu Tang.

**Supervision:** Zhan-Sheng Zhao.

**Visualization:** Zhan-Sheng Zhao, Na Li.

**Writing – original draft:** Gui-Zhi Li.

**Writing – review & editing:** Gui-Zhi Li, Zhan-Sheng Zhao.
